# Robust and dynamic underwater adhesives enabled by catechol-functionalized poly(disulfides) network

**DOI:** 10.1093/nsr/nwac139

**Published:** 2022-07-25

**Authors:** Chen-Yu Shi, Dan-Dan He, Qi Zhang, Fei Tong, Zhao-Tao Shi, He Tian, Da-Hui Qu

**Affiliations:** Key Laboratory for Advanced Materials and Joint International Research Laboratory of Precision Chemistry and Molecular Engineering, Feringa Nobel Prize Scientist Joint Research Center, Frontiers Science Center for Materiobiology and Dynamic Chemistry, School of Chemistry and Molecular Engineering, East China University of Science and Technology, Shanghai 200237, China; Key Laboratory for Advanced Materials and Joint International Research Laboratory of Precision Chemistry and Molecular Engineering, Feringa Nobel Prize Scientist Joint Research Center, Frontiers Science Center for Materiobiology and Dynamic Chemistry, School of Chemistry and Molecular Engineering, East China University of Science and Technology, Shanghai 200237, China; Key Laboratory for Advanced Materials and Joint International Research Laboratory of Precision Chemistry and Molecular Engineering, Feringa Nobel Prize Scientist Joint Research Center, Frontiers Science Center for Materiobiology and Dynamic Chemistry, School of Chemistry and Molecular Engineering, East China University of Science and Technology, Shanghai 200237, China; Key Laboratory for Advanced Materials and Joint International Research Laboratory of Precision Chemistry and Molecular Engineering, Feringa Nobel Prize Scientist Joint Research Center, Frontiers Science Center for Materiobiology and Dynamic Chemistry, School of Chemistry and Molecular Engineering, East China University of Science and Technology, Shanghai 200237, China; Key Laboratory for Advanced Materials and Joint International Research Laboratory of Precision Chemistry and Molecular Engineering, Feringa Nobel Prize Scientist Joint Research Center, Frontiers Science Center for Materiobiology and Dynamic Chemistry, School of Chemistry and Molecular Engineering, East China University of Science and Technology, Shanghai 200237, China; Key Laboratory for Advanced Materials and Joint International Research Laboratory of Precision Chemistry and Molecular Engineering, Feringa Nobel Prize Scientist Joint Research Center, Frontiers Science Center for Materiobiology and Dynamic Chemistry, School of Chemistry and Molecular Engineering, East China University of Science and Technology, Shanghai 200237, China; Key Laboratory for Advanced Materials and Joint International Research Laboratory of Precision Chemistry and Molecular Engineering, Feringa Nobel Prize Scientist Joint Research Center, Frontiers Science Center for Materiobiology and Dynamic Chemistry, School of Chemistry and Molecular Engineering, East China University of Science and Technology, Shanghai 200237, China

**Keywords:** adhesives, supramolecular materials, dynamic polymers, non-covalent crosslink, iron-catechol complexes

## Abstract

Developing molecular approaches to the creation of robust and water-resistant adhesive materials promotes a fundamental understanding of interfacial adhesion mechanisms as well as future applications of biomedical adhesive materials. Here, we present a simple and robust strategy that combines natural thioctic acid and mussel-inspired iron-catechol complexes to enable ultra-strong adhesive materials that can be used underwater and simultaneously exhibit unprecedentedly high adhesion strength on diverse surfaces. Our experimental results show that the robust crosslinking interaction of the iron-catechol complexes, as well as high-density hydrogen bonding, are responsible for the ultra-high interfacial adhesion strength. The embedding effect of the hydrophobic solvent-free network of poly(disulfides) further enhances the water-resistance. The dynamic covalent poly(disulfides) network also makes the resulting materials reconfigurable, thus enabling reusability via repeated heating and cooling. This molecule-engineering strategy offers a general and versatile solution to the design and construction of dynamic supramolecular adhesive materials.

## Teaser

A robust underwater adhesive material is achieved by coupling natural thioctic acid with mussel-inspired iron-catechol chemistry, showing a high-strength, tunable and reusable adhesion to diverse surfaces.

## INTRODUCTION

Enabling robust interfacial adhesion via human-made adhesive materials has become an attractive topic because it offers many contemporary opportunities in multidisciplinary fields, such as underwater engineering adhesives [1–3], bio-adhesion materials [4–6] and wearable devices [7,8]. Despite the exploitation and application of many commercial adhesives, a major challenge hindering the further application of current adhesive materials is the lack of strength and durability in the presence of interfacial water, which can weaken or even hinder the interfacial adhesion of many adhesive materials because of the lubricating effect of water molecules in the adhesive network. In the effort to overcome this drawback, mussels, natural masters of attaching to solid surfaces in the sea, have been in the spotlight since 1981 [9]. Mussels’ unique underwater adhesion ability is brought about by adhesive proteins that are abundantly decorated with catechol moieties, i.e. 3,4-dihydroxyphenylalanine (DOPA) [10,11]. Mussel-inspired DOPA chemistry has become a typical supramolecular toolbox for designing water-resistant adhesive materials, and has resulted in elegant progress based on the scaffold of (hydro)gels [12,13]. However, very few studies have focused on the DOPA-functionalized solvent-free network [14,15], which may enrich the supramolecular binding of DOPA moieties to the surface by avoiding solvation competition, and further boost the material performance of this bio-inspired adhesive material.

Our group recently developed a series of solvent-free supramolecular networks by using thioctic acid (TA) [16–24], a natural small molecule, as the feedstock. The reversible ring-opening polymerization (ROP) mediated by dynamic covalent disulfide bonds, together with the non-covalent crosslinking of sidechains, jointly endows the resulting network with many intriguing dynamic functions, including mechanical adaptiveness, self-healing ability, re-processability and chemical closed-loop recyclability. Meanwhile, the resulting polymeric materials also show excellent adhesive properties [19], which may be attributed to (i) high interfacial penetration due to the small-molecule precursor, (ii) the high abundance of hydrogen bonds in the carboxylic sidechain and (iii) the solvent-free nature of the network. Motivated by these features, we envisioned that it would be exciting if one could introduce mussel-inspired DOPA chemistry into the solvent-free network of poly(TA) so that the advantages of both adhesion chemistries could be combined in a single material that is a high-strength adhesive, while simultaneously being water-resistant, durable and dynamic.

Here we report an unprecedentedly robust underwater adhesive material that incorporates TA-based dynamic polymers and mussel-inspired adhesion chemistry. By modifying the TA sidechain with a catechol unit to obtain thioctic acylamino catechol (TAC) monomers, the copolymer network of TA and TAC can be readily prepared and controlled by one-pot solvent-free ROP. The optimized supramolecular network exhibits dynamic properties as well as a high-strength adhesion ability (shear strength >11 MPa). The robust interfacial adhesion shows excellent water-resistance, durability and reusability. Also, considering the biocompatibility of TA and the many dynamic features of this material [25], we anticipate that this design strategy and its related materials will provide many opportunities in the design and application of wearable electronics and bio-adhesive materials.

## RESULTS AND DISCUSSION

### Preparation and characterization

**Figure 1.**
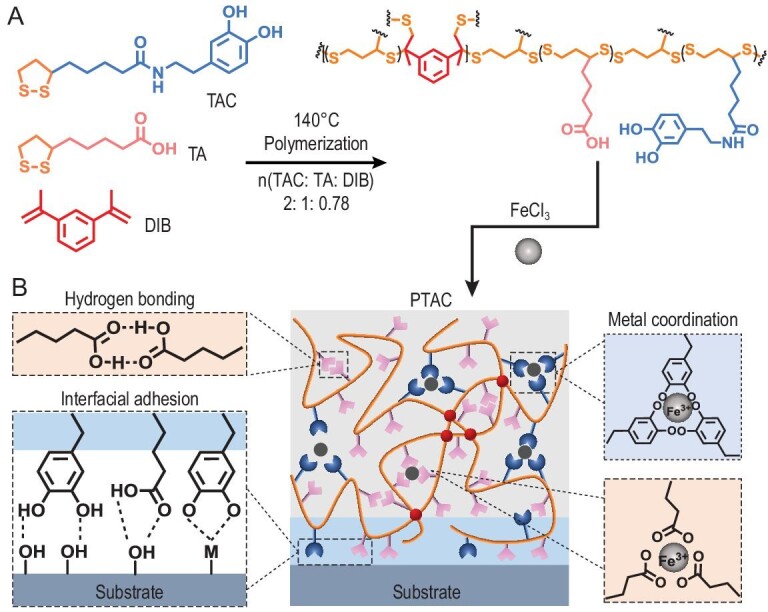
Schematic representation of (A) the monomer structures, synthesis conditions and polymer networks, and (B) the cohesive networks and interface adhesion mechanism of the PTAC copolymer.

To modify the TA sidechain with catechol unit, a one-step amide coupling reaction was used to prepare monomer TAC (Scheme S1, Figs S1 and S2). Then the TAC monomer could be further polymerized or copolymerized with TA, in the presence of covalent crosslinker 1,3-diisopropenylbenzene (DIB) and Fe (III) ion, via a one-pot solvent-free melting polymerization method (Fig. 1A) [18,19]. The optimized ratio of TAC:TA:DIB:Fe (III) was set as 1:2:0.78:0.33 for maximal adhesion strength on dry substrates over 10 MPa (Fig. S3) and 2:1:0.78:0.1 for maximal underwater adhesion strength exceeding 5 MPa. The resulting copolymers exhibited high tensile strength and stretchability, and the macroscopic properties could be readily controlled by the structures and molar ratios of precursor monomers (Fig. 1B). For comparison, two reference analogues were synthesized by replacing catechol units with phenol (TAP) and benzene (TAB) groups, respectively (Figs S4–S7).

**Figure 2.**
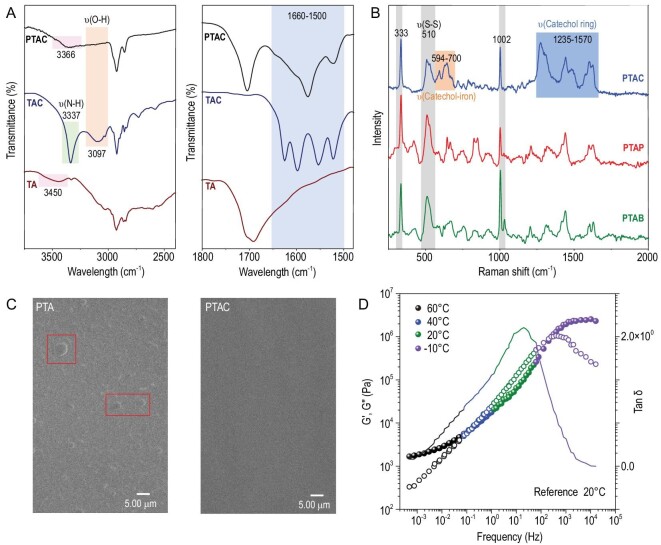
The characterization of the polymer structures. (A) Partial FT-IR spectra of TA monomer, TAC monomer and PTAC copolymer. (B) Raman spectra of the PTAC, PTAP and PTAB copolymer. (C) SEM images of the PTA and PTAC copolymer (scale bar is 5.00 μm). (D) Master curves of the PTAC copolymer from rheological properties at a reference temperature of 20^o^C. (The frequency sweep curves above 70°C failed to match the TTS due to the dissociation of heat-labile iron-catechol complexes.)

Spectroscopic characterizations, which determine the main adhesive behavior of a copolymer, were conducted to investigate the formation of iron-catechol complexes. The attenuated-total-reflection Fourier transformed infrared (ATR-FTIR) spectra of poly(thioctic acylamino catechol) (PTAC) showed a small new broad peak near 3366 cm^−1^ (Fig. 2A). Compared with the high sharp single peak at ∼3337 cm^−1^ in TAC and a small peak at ∼3450 cm^−1^ of TA, this new peak suggested the formation of abundant associated H-bonds in the polymer networks. In addition, the disappearance of the peak in TAC at 3097 cm^−1^ corresponded to the phenolic hydroxyl vibration, and the broad peaks in PTAC from 1660 to 1500 cm^−1^ were attributed to the vibration of benzene rings and indicated the formation of iron-catechol complexes [26,27]. The formation of iron-catechol complexes was also revealed by Raman spectroscopy, by the distinctive peaks from 594 to 700 cm^−1^ and 1235 to 1570 cm^−1^ in PTAC, which were attributed to the vibration iron-catechol bond and catechol ring, respectively (Fig. 2B) [28,29].

Scanning electron microscopy (SEM) was used to image the microscopic morphology of the resulting network, showing the existence of iron aggregates of PTA but no visible aggregates or particles in the case of PTAC (Figs 2>C and S8), suggesting the homogeneity of the iron-catechol complexes in the PTAC network. Further characterization results by X-ray diffraction (XRD) and small-angle X-ray scattering (SAXS) techniques showed the amorphous nature of the PTAC, poly(thioctic acylamino phenol) (PTAP) and poly(thioctic acylamino benzene) (PTAB) network (Fig. S9), whereas some sharp diffraction peaks were observed in the PTA network. Small-amplitude oscillatory shear (SAOS) measurements were performed to investigate the bulky properties of PTAC, and master curves from rheological properties at the reference temperature of 20°C (room temperature) were obtained via time-temperature superposition (TTS) (Figs 2D and S10). The curves exhibited dynamic viscoelastic behavior typical of amorphous polymers [30]. The high-frequency region (>10^2^ Hz) with G′ > G″ corresponded to the glassy state of the PTAC where the polymer networks were inactive. Below the transition point where G′ < G″ (10^−1^–10^2^ Hz), the region was identified as the dissipative regime manifesting active alternation of supramolecular chain segments. The low frequency range with G′ > G″ (10^–3^–10^−1^ Hz) corresponded to the Rouse dynamics of the PTAC chains.

### Underwater adhesion properties

**Figure 3.**
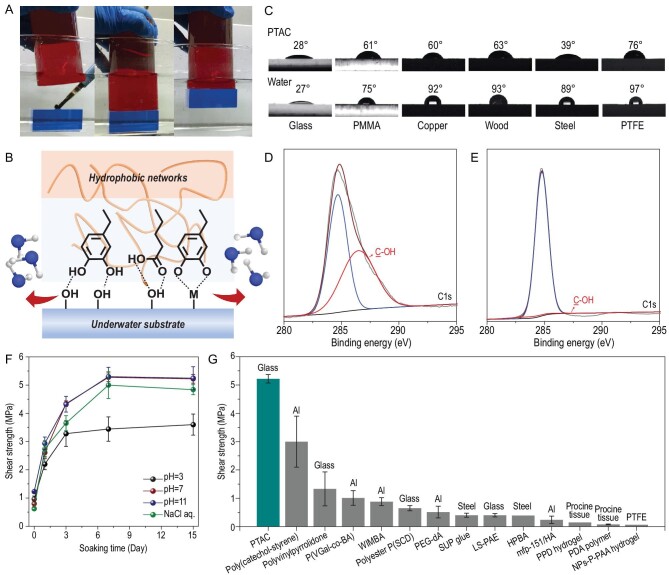
(A) A photograph of the instant adhesion of two glass blocks coated with PTAC copolymer adhesive in deionized water. (B) Schematic illustration of the underwater adhesion mechanism. (C) Contact angles of PTAC copolymer and water on different substrates (including glass, PMMA, copper, wood, steel and PTFE). (D) Peak-fitting XPS spectra in the C1s regions of the PTAC copolymer. (E) Peak-fitting XPS spectra in the C1s regions of the lyophilized PTAC copolymer immersed in water. (F) Time-dependent adhesion strength curves of the PTAC copolymer soaking in different water environments. (G) Comparison of underwater adhesion strength of the PTAC copolymer with other reported bio-inspired underwater adhesives. Reproduced with permission from refs [15,31–42].

Then we moved to the experimental evaluation of the adhesion performance in dry and wet environments. Systematic experiments showed that the optimal materials for underwater adhesion had a TAC:TA:DIB:Fe (III) molar ratio of 2:1:0.78:0.1 (Fig. S11). The resulting copolymer provided flexible polymeric networks and more hydrogen bonding sites to overcome the barrier of the hydration layer. Therefore, the PTAC copolymer exhibited robust water-resistant adhesion to a variety of substrates even in the presence of interfacial water (Fig. S12). A glass block (500 g) coated with 10 μL PTAC copolymer adhesive in deionized water can be easily lifted up by another glass block (∼2 cm^2^ contact area) (Fig. 3A), indicating the robust interfacial adhesion mediated by non-covalent bonds between the PTAC network and substrates (Fig. 3B). The unusual water-resistant interfacial adhesion ability suggests that the hydrophobic but polar PTAC network may induce interfacial water replacement upon adhesion, thus resulting in robust underwater adhesion. To support this hypothesis, the contact angles of the molten PTAC copolymer and water on various substrates were measured (Fig. 3C). As a result, PTAC copolymer liquid exhibited lower surface tension than water on different types of substrates, including glass, polymethyl methacrylate (PMMA), copper, wood, steel and polytetrafluoroethylene (PTFE), indicating the higher surface affinity of PTAC polymer than water molecules. Meanwhile, surface element analysis by X-ray photoelectron spectroscopy (XPS) revealed that the contents of C-OH on the dry surface of PTAC accounted for 47.54% of all C elements (Fig. 3D), while the surface proportion of C-OH was decreased to only 2.86% of the C elements (Fig. 3E) when PTAC was immersed in water for 5 min. These observations jointly indicated the tough bonding capability of high-density hydrophilic catechol groups on the surface, and the prominent waterproof features of polymer skeletons.

Quantitative lap shear tests were conducted after the PTAC copolymer was adhered to glass slices underwater and placed in water environments for different time intervals. The PTAC copolymer immediately adhered to the glass substrates, and the instant adhesion strength reached 0.8 MPa (Fig. 3F). The strength of adhesion increased dramatically as the solidification time increased, and ultimately stabilized at 5.3 MPa as the curing time extended to 7 days. To the best of our knowledge, this is the state-of-the-art underwater adhesive material, with regard to strength and durability (Fig. 3G) [15,31–42]. The suspension measurements showed that a 5-kg weight could be hung for over 10 days when placed under just two adhered glass slices with a glued area of 2 × 2 cm^2^ underwater (Fig. S13). A persistently improved adhesion strength was ascribed to the further crosslinking of unassociated non-covalent bonds, which significantly toughened the aging cohesive networks.

Towards more practical applications in different underwater conditions, the adhesion performance of PTAC was tested in different water environments for different time periods, including artificial seawater and aqueous solutions over a wide pH range from 3 to 11 (Fig. 3F). The adhesion strength of these samples in different aqueous environments increased with longer solidification time, and then showed stabilized adhesion strength after 7 days. The adhesion strength at pH 11 and in NaCl aqueous solution was nearly consistent with the value at pH 7. At pH 3, the partial loss of adhesion performance could be due to the acid-induced dissociation of metal-catechol complexes and H-bonds. Even so, the adhesion strength remained at a moderate level up to 3.6 MPa, indicating durability in acidic environments.

### Mechanical properties and dry adhesion behaviors

**Figure 4.**
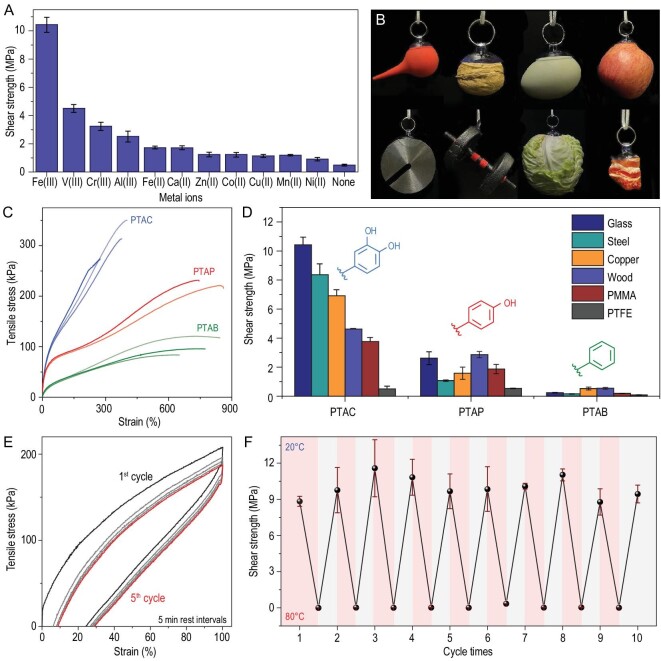
(A) The shear strength of PTAC copolymer adhered to glass, combining diverse metal complexes. (B) Digital images of the PTAC adhesives suspending a wide range of objects. (C) Stress-strain curves of the PTAC, PTAP and PTAB copolymers. (D) Comparison of shear strength among the PTAC, PTAP and PTAB copolymers on different substrates. (E) Five loading–unloading cycles of the PTAC copolymer with 5 min rest intervals at a loading rate of 20 mm min^−1^. (F) The shear strength curves of the PTAC copolymer after 10 temperature-cycling experiments.

For dry interfacial adhesion, the amount of Fe (III) was set as a 33% molar ratio of monomer TAC, forming high-strength iron-catechol complexes as rigid domains, but excess iron-catechol complexes led to brittle polymeric networks and adhesion failure (Fig. S3). Copolymerization with TA monomers led to effective synergy between strong iron-catechol complexes and weak H-bonding crosslinks, thus exhibiting optimized adhesion strength. The molar ratio of TAC:TA:DIB:Fe (III) was set as 1:2:0.78:0.33. The hot-melting copolymer liquid was first dropped on a glass sheet and hot-pressed by another glass sheet for further adhesion measurements. Lap shear tests of PTAC copolymers combining diverse metal complexes (including Fe (III), V (III), Cr (III), Al (III), Fe (II), Ca (II), Zn (II), Co (II), Cu (II), Mg (II) and Ni (II)) were conducted to verify that the coordination intensity largely affected the adhesion capacity; the iron-coordinated copolymer exhibited a maximal shear strength up to 11.7 MPa (Fig. 4A). Quantitative tests further demonstrated the effects of the molar ratio of Fe (III):TAC on adhesion strength. The shear strength increased with the molar ratio of Fe (III):TAC, suggesting the key role of iron-catechol complexes in adhesion toughness (Fig. S14). PTAC was not limited to smooth and dry substrates. It could directly adhere to a wide range of objects that had rough and uneven wet surfaces (Fig. 4B), showing great potential for practical applications.

To verify the regulation of precursor molecular structure on macroscopic properties of the ensembles, we next conducted a series of thermal and mechanical property measurements of PTAC, PTAP and PTAB. The glass transition temperature (Tg) of PTAC was higher than that of PTAP and PTAB reference copolymers due to the high-intensity iron-catechol complexes (Figs S15 and S16) in the polymer network. The interaction distinction in the microscopic polymeric networks could be further amplified to different mechanical properties at the macroscopic level. PTAC exhibited superior mechanical robustness relative to the other two references (i.e. PTAP and PTAB). Figure 4C shows that the tensile stress of PTAC (331.9 ± 18.3 kPa) was ∼1.5-fold the tensile stress of PTAP (226.3 ± 5.3 kPa), and nearly 3.4-fold the tensile stress of PTAB (99.0 ± 14.0 kPa). Moreover, the Young's modulus of PTAC (492.3 ± 1.1 kPa) was ∼1.7-fold that of PTAP (292.3 ± 12.9 kPa), and more than one order of magnitude greater than PTAB (48.5 ± 1.8 kPa, Fig. S17). Meanwhile, the elongation of PTAC was partially lost due to the dense coordination that weakened the fluidity of the polymer chains. The PTAC copolymer also exhibited higher adhesion strength on six different substrates compared to PTAP and PTAB copolymers because of the enhanced interfacial H-bonding sites between the substrate and copolymer network (Fig. 4D). The perpetual cyclic loading–unloading process would lead to residual strain, originating from the breaking of coordinate bonds during elongation and their reformation at new sites (Fig. S18). The complete recovery of elastic modulus was observed after a longer resting interval of 5 min, suggesting striking features of fast kinetics and dynamic adaptivity bonding in the supramolecular polymer (Fig. 4E). The dynamic characteristics of networks were further confirmed by stress-relaxation tests, and the instantaneous stress at 10% strain was 2.7 MPa. This subsequently relaxed to 1.5 kPa within 2 h, which indicates a fast dynamic bond exchange motion under external forces (Fig. S19). The creep recovery curves showed that the resulting amorphous polymeric networks could be sustainable, with a load, for a long period and recover to the original state rapidly after the load was removed (Figs S20 and S21).

### Applications as dynamic recyclable materials

Intrinsically dynamic and reconfigurable poly(disulfide) backbones offered additional opportunities as recyclable polymer materials. They can achieve self-healing, on-demand (de)bonding and reusable 3D printing. The cut gaps nearly disappeared under the optical microscope after being left at ambient temperature for 24 h, which suggests that autonomous self-healing occurred in the PTAC copolymer (Fig. S22). Continuous strain sweep variation experiments were performed to further monitor the self-recovery degree of supramolecular networks. Low oscillation strain (0.1%) and high oscillation strain (40%) were applied to measure storage modulus (G′) and loss modulus (G″) values alternately (Fig. S23). The G′ of PTAC copolymer was constantly ∼1.2 × 10^4^ Pa when applied to low strain, and the G′ value dropped to 7.0 × 10^3^ Pa when applied to high strain. This showed a breakdown of crosslinked networks. The G′ could regain the original value after five cycles of strain sweep variation, demonstrating that the fast self-healing of damaged networks is attributed to the synergy of three kinds of dynamics bonding, including disulfide bonding, metal-coordination and H-bonding.

The temperature-cycle rheology curves showed a significant change and lossless recovery of the modulus of the PTAC copolymer with temperature due to the thermo-sensitive weak interactions (Fig. S24). Therefore, the shear strength of PTAC decreased to 0.01 MPa and the adhesion tended to decline under 80°C. The separated substrates again adhered when the temperature was cooled to 20°C because of the reformation of interfacial H-bonds. The adhesion performance showed no fatigue over 10 cycles (Fig. 4F). The excellent reusability and processability meant that the PTAC copolymer was easily hot-extruded and rapidly cool-molded. Thus, it could become a potential candidate for 3D-printing raw materials with specific shapes and sizes (Fig. S25).

## CONCLUSION

In summary, we successfully demonstrate a high-performance supramolecular adhesive material based on PTAC copolymer with instant superior adhesion capacity in various environments (including dry conditions, deionized water, seawater and a wide range of pH (3 to 11) solutions). The excellent adhesion properties of PTAC were ascribed to the combination of dynamic hydrophobic polymeric backbones, high-density hydroxy catechol groups and strong iron-catechol coordination. In addition, the intrinsic dynamic poly(disulfide) backbones gave the copolymer self-healing, on-demand (de)bonding and reusable 3D printing. The novel hot melt adhesive shows great potential in many fields, including automotive, electronics and packaging industries, owing to its solvent-free nature and capability of forming adhesion rapidly with diverse substrates. We expect that the unique supramolecular self-assembly strategy will lead to more possibilities of bio-inspired adhesive materials both in molecular engineering and performance extension.

## METHODS

**Preparation of PTAC:** TAC (5 g, 14.6 mmol) and TA (6 g, 29.3 mmol) monomer powder was added to a flask and then heated in an oil bath (140^o^C) to form a yellow transparent low-viscosity molten liquid. DIB (1.8 g, 11.4 mmol) was added into the liquid by injection and further stirred at 140°C for 5 min. Then a given amount of FeCl_3_·6H_2_O (1.3 g, 4.8 mmol) was dissolved in a minimal amount of acetone, which was added to the liquid mixture under vigorous stirring. Finally, a dark brown solid copolymer was obtained, after cooling down to room temperature, for further characterization and adhesive tests on dry substrates [n (TAC:TA:DIB:FeCl_3_·6H_2_O) = 1:2:0.78:0.33].

The preparation process of the PTAC copolymer for underwater adhesive tests was the same as the above method [n (TAC:TA:DIB:FeCl_3_·6H_2_O) = 2:1:0.78:0.1].

The preparation process of the PTAP copolymer was the same as the above method [n (TAP:TA:DIB:FeCl_3_·6H_2_O) = 1:2:0.78:0.33].

The preparation process of the PTAB copolymer was the same as the above method [n (TAB:TA:DIB:FeCl_3_·6H_2_O) = 1:2:0.78:0.33].

The preparation process of the PTA copolymer was the same as the above method [n (TA:DIB:FeCl_3_·6H_2_O) = 3:0.78:0.33].

**Methods for adhesive tests:** The low-viscosity hot liquid polymer was deposited onto a substrate. The deposition area was fixed as 1.0 cm × 1.0 cm. After the deposition, another substrate was used to hot-press onto the deposited liquid polymer. The shear strength experiments were performed on an HY-0580 tension machine (HENGYI Company). The two substrates adhered between the two fixtures in a vertical direction. The strain rate was 20 mm min^−1^, and the data were recorded in real time.

**Methods for mechanical test:** All stress-strain curves were obtained from a HY-0580 tension machine (HENGYI Company). The cylindrical-shaped tested samples (height, 20 mm; diameter, 4.72 mm) were obtained via molding in plastic injection syringes. The initial length was controlled at 10 mm. Unless otherwise noted, the tensile stress was measured at a constant speed of 20 mm min^−1^. The data were recorded in real time by a connected computer.

## Supplementary Material

nwac139_Supplemental_File

## References

[1] Ahn Y , Jang Y, Selvapalam N et al. Supramolecular velcro for reversible underwater adhesion. Nat Commun 2013; 52: 711–9. 10.1002/anie.201209382 23382064

[2] Hofman AH , Hees IAV, Yang J et al. Bioinspired underwater adhesives by using the supramolecular toolbox. Adv Mater 2018; 30: 1704640. 10.1002/adma.201704640 29356146

[3] Li X , Deng Y, Lai J et al. Tough, long-term, water-resistant, and underwater adhesion of low-molecular-weight supramolecular adhesives. J Am Chem Soc 2020; 142: 5371–9. 10.1021/jacs.0c00520 32092262

[4] He W , Wang Z, Hou C et al. Mucus-inspired supramolecular adhesives with oil-regulated molecular configurations and long-lasting antibacterial properties. ACS Appl Mater Interfaces 2020; 12: 16877–86. 10.1021/acsami.0c00531 32191026

[5] Yang J , Zhang G, Wang J et al. Parthenocissus-inspired, strongly adhesive, efficiently self-healing polymers for energetic adhesive applications. J Mater Chem A 2021; 9: 16076–85. 10.1039/D1TA03885K

[6] Shi C , Zhang Q, Tian H et al. Supramolecular adhesive materials from small-molecule self-assembly. SmartMat 2020; 1: e1012. 10.1002/smm2.1012

[7] Hammock ML , Chortos A, Tee BCK et al. 25th anniversary article: the evolution of electronic skin (E-skin): a brief history, design considerations, and recent progress. Adv Mater 2013; 25: 5997–6038. 10.1002/adma.201302240 24151185

[8] Li T , Wang Y, Li S et al. Mechanically robust, elastic, and healable ionogels for highly sensitive ultra-durable ionic skins. Adv Mater 2020; 32: 2002706. 10.1002/adma.202002706 32589326

[9] Waite JH , Tanzer ML. Polyphenolic substance of mytilus edulis: novel adhesive containing L-dopa and hydroxyproline. Science 1981; 212: 1038–40. 10.1126/science.212.4498.1038 17779975

[10] Maier GP , Rapp MV, Waite JH et al. Adaptive synergy between catechol and lysine promotes wet adhesion by surface salt displacement. Science 2015; 349: 628–32. 10.1126/science.aab0556 26250681

[11] Wilker JJ . Positive charges and underwater adhesion. Science 2015; 349: 582–3. 10.1126/science.aac8174 26250668

[12] Hofman AH , Hees IAV, Yang J et al. Bioinspired underwater adhesives by using the supramolecular toolbox. Adv Mater 2018; 30: 1704640. 10.1002/adma.201704640 29356146

[13] Zhang W , Wang R, Sun ZM et al. Catechol-functionalized hydrogels: biomimetic design, adhesion mechanism, and biomedical applications. Chem Soc Rev 2020; 49: 433–64. 10.1039/C9CS00285E 31939475 PMC7208057

[14] Yi B , Liu P, Hou C et al. Dual-cross-linked supramolecular polysiloxanes for mechanically tunable, damage-healable and oil-repellent polymeric coatings. ACS Appl Mater Interfaces 2019; 11: 47382–9. 10.1021/acsami.9b17199 31746582

[15] Cui C , Fan C, Wu Y et al. Water-triggered hyperbranched polymer universal adhesives: from strong underwater adhesion to rapid sealing hemostasis. Adv Mater 2019; 31: 1905761. 10.1002/adma.201905761 31625635

[16] Zhang Q , Deng YX, Luo HX et al. Assembling a natural small molecule into a supramolecular network with high structural order and dynamic functions. J Am Chem Soc 2019; 141: 12804–14. 10.1021/jacs.9b05740 31348651 PMC6696886

[17] Zhang Q , Deng Y, Shi CY et al. Dual closed-loop chemical recycling of synthetic polymers by intrinsically reconfigurable poly(disulfides). Matter 2020; 4: 1352–64. 10.1016/j.matt.2021.01.014

[18] Deng YX , Zhang Q, Feringa BL et al. Toughening a self-healable supramolecular polymer by ionic cluster-enhanced iron-carboxylate complexes. Angew Chem Int Ed 2020; 59: 5278–83. 10.1002/anie.201913893 32096593

[19] Zhang Q , Shi CY, Qu DH et al. Exploring a naturally tailored small molecule for stretchable, self-healing, and adhesive supramolecular polymers. Sci Adv 2018; 4: eaat8192. 10.1126/sciadv.aat8192 30062126 PMC6063538

[20] Shi CY , Zhang Q, Wang BS et al. Intrinsically photopolymerizable dynamic polymers derived from a natural small molecule. ACS Appl Mater Interfaces 2021; 13: 44860–7. 10.1021/acsami.1c11679 34499480

[21] Zhang Q , Crespi S, Toyoda R et al. Stereodivergent chirality transfer by noncovalent control of disulfide bonds. J Am Chem Soc 2022; 144: 4376–82. 10.1021/jacs.1c10000 35120292 PMC8931715

[22] Zhang Q , Qu DH, Feringa BL et al. Disulfide-mediated reversible polymerization toward intrinsically dynamic smart materials. J Am Chem Soc 2022; 144: 2022–33. 10.1021/jacs.1c10359 34990126

[23] Deng YX , Zhang Q, Shi CY et al. Acylhydrazine-based reticular hydrogen bonds enable robust, tough and dynamic supramolecular materials. Sci Adv 2022; 8: eabk3286. 10.1126/sciadv.abk3286 35089796 PMC8797780

[24] Sieredzinska B , Zhang Q, Berg KJ et al. Photo-crosslinking polymers by dynamic covalent disulfide bonds. Chem Commun 2021; 57: 9838–41. 10.1039/D1CC03648C PMC8477374 34498635

[25] Packer L , Witt EH, Tritschler HJ. Alpha-lipoic acid as a biological antioxidant. Free Radicals Biol Med 1995; 19: 227–50. 10.1016/0891-5849(95)00017-R 7649494

[26] Tang Z , Zhao M, Wang Y et al. Mussel-inspired cellulose-based adhesive with biocompatibility and strong mechanical strength via metal coordination. Int J Biol Macromol 2020; 144: 127–34. 10.1016/j.ijbiomac.2019.12.076 31837365

[27] He Y , Gao S, Jubsilp C et al. Reprocessable polybenzoxazine thermosets crosslinked by mussel-inspired catechol-Fe^3+^ coordination bonds. Polymer 2020; 192: 122307. 10.1016/j.polymer.2020.122307

[28] Filippidi E , Cristiani TR, Eisenbach CD et al. Toughening elastomers using mussel-inspired iron-catechol complexes. Science 2017; 358: 502–5. 10.1126/science.aao0350 29074770 PMC5676464

[29] Harrington MJ , Masic A, Holten-Andersen N et al. Iron-clad fibers: a metal-based biological strategy for hard flexible coatings. Science 2010; 328: 216–20. 10.1126/science.1181044 20203014 PMC3087814

[30] Yanagisawa Y , Nan Y, Okuro K et al. Mechanically robust, readily repairable polymers via tailored noncovalent cross-linking. Science 2018; 359: 72–6. 10.1126/science.aam7588 29242235

[31] North MA , Grosso CAD, Wilker JJ. High strength underwater bonding with polymer mimics of mussel adhesive proteins. ACS Appl Mater Interfaces 2017; 9: 7866–72. 10.1021/acsami.7b00270 28177600

[32] Li A , Mu Y, Jiang W et al. A mussel-inspired adhesive with stronger bonding strength under underwater conditions than under dry conditions. Chem Commun 2015; 51: 9117–20. 10.1039/C5CC00101C 25940100

[33] Zhan K , Kim C, Sung K et al. Tunicate-inspired gallol polymers for underwater adhesive: a comparative study of catechol and gallol. Biomacromolecules 2017; 18: 2959–66. 10.1021/acs.biomac.7b00921 28853566

[34] Kim HJ , Hwang BH, Lim S et al. Mussel adhesion-employed water-immiscible fluid bioadhesive for urinary fistula sealing. Biomaterials 2015; 72: 104–11. 10.1016/j.biomaterials.2015.08.055 26352517

[35] Xu Y , Liu Q, Narayanan A et al. Mussel-inspired polyesters with aliphatic pendant groups demonstrate the importance of hydrophobicity in underwater adhesion. Adv Mater Interfaces 2017; 4: 1700506. 10.1002/admi.201700506

[36] Kaur S , Weerasekare GM, Stewart RJ. Multiphase adhesive coacervates inspired by the sandcastle worm. ACS Appl Mater Interfaces 2011; 3: 941–4. 10.1021/am200082v 21410239 PMC3083470

[37] Sun J , Xiao L, Li B et al. Genetically engineered polypeptide adhesive coacervates for surgical applications. Angew Chem Int Ed 2021; 60: 23687–94. 10.1002/anie.202100064 PMC8596419 33886148

[38] Wei C , Zhu X, Peng H et al. Facile preparation of lignin-based underwater adhesives with improved performances. ACS Sustain Chem Eng 2019; 7: 4508–14. 10.1021/acssuschemeng.8b06731

[39] Lim S , Choi YS, Kang DG et al. The adhesive properties of coacervated recombinant hybrid mussel adhesive proteins. Biomaterials 2010; 31: 3715–22. 10.1016/j.biomaterials.2010.01.063 20144475

[40] Wang R , Li J, Chen W et al. A biomimetic mussel-inspired ϵ-poly-L-lysine hydrogel with robust tissue-anchor and anti-infection capacity. Adv Funct Mater 2017; 27: 1604894. 10.1002/adfm.201604894

[41] Zhang H , Bré LP, Zhao T et al. Mussel-inspired hyperbranched poly(amino ester) polymer as strong wet tissue adhesive. Biomaterials 2014; 35: 711–9. 10.1016/j.biomaterials.2013.10.017 24140046

[42] Gan D , Xin W, Jiang L et al. Plant-inspired adhesive and tough hydrogel based on Ag-Lignin nanoparticles-triggered dynamic redox catechol chemistry. Nat Commun 2019; 10: 1487. 10.1038/s41467-019-09351-2 30940814 PMC6445137

