# Supplementary material for: Robust and dynamic underwater adhesives enabled by catechol-functionalized poly(disulfides) network
Source: Natl Sci Rev. 2022 Jul 25;10(2):nwac139. doi: 10.1093/nsr/nwac139 (PMC10042223; doi:10.1093/nsr/nwac139)
Supplement: nwac139_Supplemental_File [file nwac139_supplemental_file.docx]

***Supporting Information***

**Robust and dynamic underwater adhesives enabled by catechol-functionalized poly(disulfides) network**

Chen-Yu Shi^1^, Dan-Dan He^1^, Qi Zhang^1^, Fei Tong^1^, Zhao-Tao Shi^1^, He Tian^1^, Da-Hui Qu^1^*

^1^Key Laboratory for Advanced Materials and Joint International Research Laboratory of Precision Chemistry and Molecular Engineering, Feringa Nobel Prize Scientist Joint Research Center, Frontiers Science Center for Materiobiology and Dynamic Chemistry, School of Chemistry and Molecular Engineering, East China University of Science and Technology, Shanghai 200237, China.

Email: Da-Hui Qu: dahui_qu@ecust.edu.cn

**Content:**

**1. Materials and methods**

**2. Supporting figures**

**Scheme S1.** The synthetic routes to compounds TAC, TAP and TAB.

**Figure S1.** ^1^H NMR spectrum of compound TAC (400 MHz, 298 K, Acetone-*d_6_*).

**Figure S2.** ESI-mass spectrum of compound TAC.

**Figure S3.** Shear strength histogram of the PTAC copolymer with different molar ratio of TA to TAC.

**Figure S4.** ^1^H NMR spectrum of compound TAP (400 MHz, 298 K, DMSO-*d_6_*).

**Figure S5.** ESI-mass spectrum of compound TAP.

**Figure S6.** ^1^H NMR spectrum of compound TAB (400 MHz, 298 K, DMSO-*d_6_*).

**Figure S7.** ESI-mass spectrum of compound TAB.

**Figure S8.** SEM images of the PTA and PTAC copolymer. (Scale bar is 2.00 μm.)

**Figure S9.** XRD and SAXS spectrum of the PTAC, PTAP and PTAB copolymers.

**Figure S10.** Temperature-dependence of viscoelastic shift factors a_T_ of master curves at a reference temperature of 20^o^C.

**Figure S11.** Underwater shear strength histogram of the PTAC copolymer with different iron (III) concentrations.

**Figure S12.** Comparison of underwater shear strength of the PTAC copolymer on different substrates.

**Figure S13.** Photograph of the PTAC copolymer bonded glass sheets to lift weights of 5 kg with a bonding area of 2×2 cm^2^.

**Figure S14.** Shear strength of the PTAC copolymer for different TAC-to-iron(III) molar ratios.

**Figure S15.** TGA of the PTAC, PTAP and PTAB copolymers.

**Figure S16.** DSC of the PTAC, PTAP and PTAB copolymers.

**Figure S17.** Young’s Modulus of the PTAC, PTAP and PTAB copolymers.

**Figure S18.** Five successive loading-unloading cycles of the PTAC copolymer at a loading rate of 20 mm min^-1^.

**Figure S19.** Stress relaxation experiment performed on the PTAC copolymer. The relaxation strain was 10%.

**Figure S20.** Creep experiments at varying stress were performed on the PTAC copolymer.

**Figure S21.** Creep recovery curves of the PTAC copolymer.

**Figure S22.** Photographs depict that scratches on the PTAC copolymer membrane can be autonomously cured after 24 hours at room temperature.

**Figure S23.** Storage (G′) and loss (G″) moduli variation under continuous strain sweep variation with small oscillation (0.1 % strain, solid line) and large oscillation (40 % strain, dot line) force alternatively. Angular frequency = 1 Hz.

**Figure S24.** Temperature-cycle rheology curves of the PTAC copolymer.

**Figure S25.** Photographs of 3D-printing with specific shapes and sizes.

**1. Materials and methods**

**General Information:** All the reagents were purchased from Adamas®beta, TCI and Aldrich. The key (±)-α-thioctic acid (TA) (Reagent Grade, 99%) was used as received from Adamas®beta. Chemicals were weighed on analytical balances (METTLER-TOLEDO, ME204T/02). Nuclear magnetic resonance (NMR) spectrum was tested by Brüker AV-400 spectrometer using tetramethylsilane to be the internal standard. The electronic spray ionization (ESI) mass spectra were obtained on XEVO G2 TOF. X-ray diffraction (XRD) patterns were obtained on a rotating anode X-ray powder diffractometer (18KW/D/max2550VB/PC) equipped with a copper target 18KW (450mA), a fully automated curved (plate) crystal graphite monochoromator and a programmed variable slit system. Field emission scanning electron microscopy (FE-SEM) (JSM-6360LV5-300000) was used to detect the surface morphology imaging of the copolymer samples. The Raman spectra were recorded by using a Laser Micro-Raman Spectrometer (Renishaw, I0.2/cm/invia reflex) equipped with a high performance grade Leica DMLM microscope and a 514 nm excitation wavelength. Fourier transform infrared spectrometer (FT-IR) (Thermo Nicolet Corporation; 7800-350/cm 0.01/cm/6700) was used to analyze the copolymer samples. The valence state of iron was determined with X-ray photoelectron spectroscopy (XPS) (Thermo Fisher Corporation; resolution ratio > 3 μm/ESCALAB 250Xi). The mechanical properties of the polymer films were measured by an HY-0580 tension machine (HENGYI), and the thermal stability was measured by thermogravimetric analysis (Mettler Toledo TGA/SDTA851, heating rate = 5°C min^−1^). The thermal properties were measured by differential scanning calorimetry (TA instruments; modulated DSC2910, 1090B). The rheological experiments were performed by rotational rheometer (TA Instruments-Waters LLC; DHR-2). The polarized optical microscopy images were obtained on a typical optical microscopy equipped with two polarizers.

**2. Supporting figures**

**Scheme S1.** The synthesis routes to compounds TAC, TAP and TAB.

**
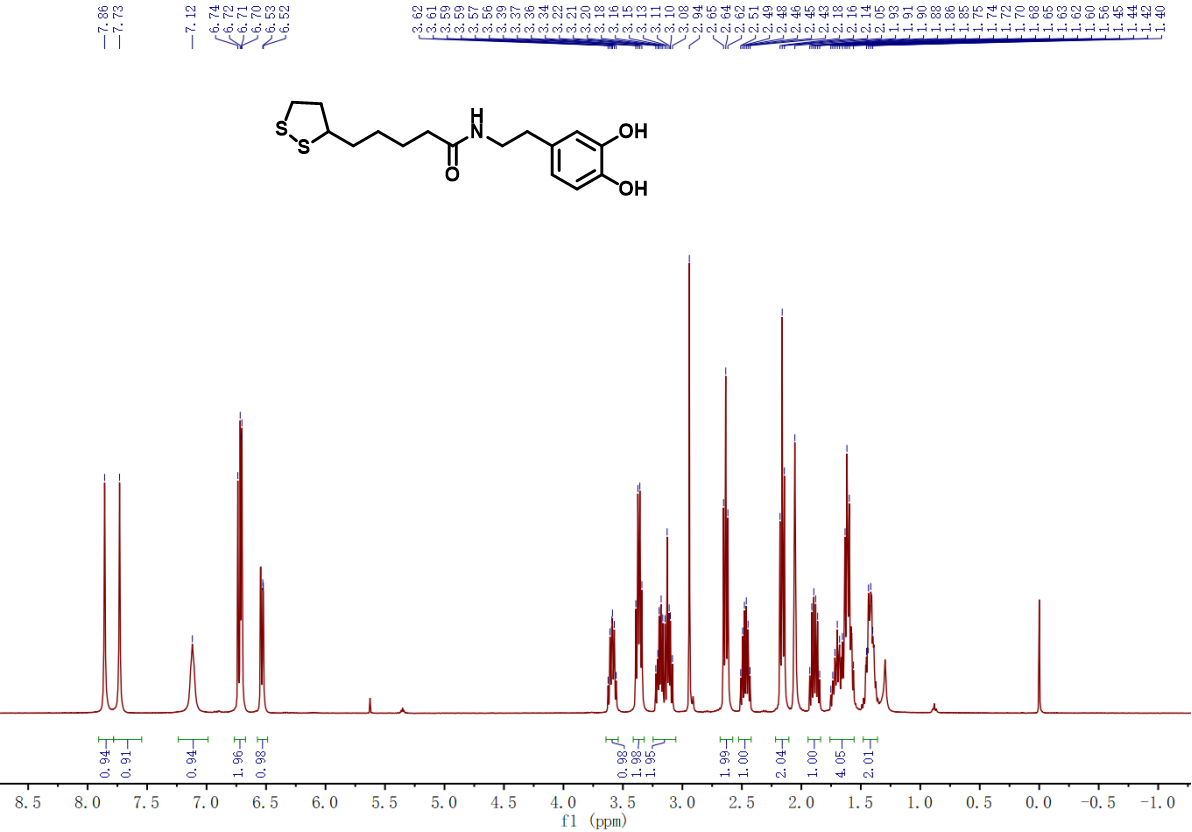
**

**Figure S1.** ^1^H NMR spectrum of compound TAC (400 MHz, 298 K, Acetone-*d_6_*).

**Figure S2.** ESI-mass spectrum of compound TAC ([M+Na^+^]^+^: 364.1019).

**
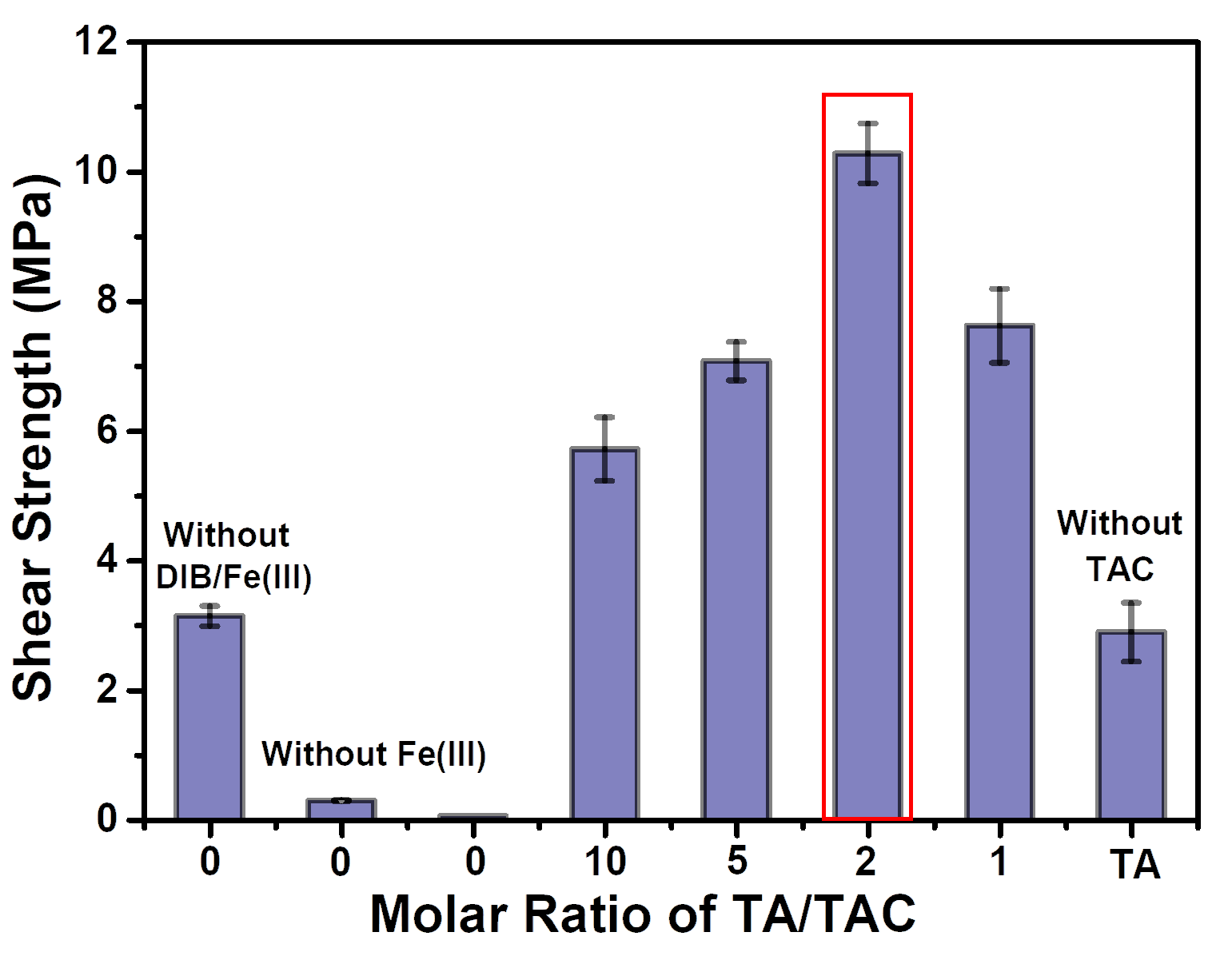
**

**Figure S3.** Shear strength histogram of the PTAC copolymer with different molar ratio of TA to TAC.

**
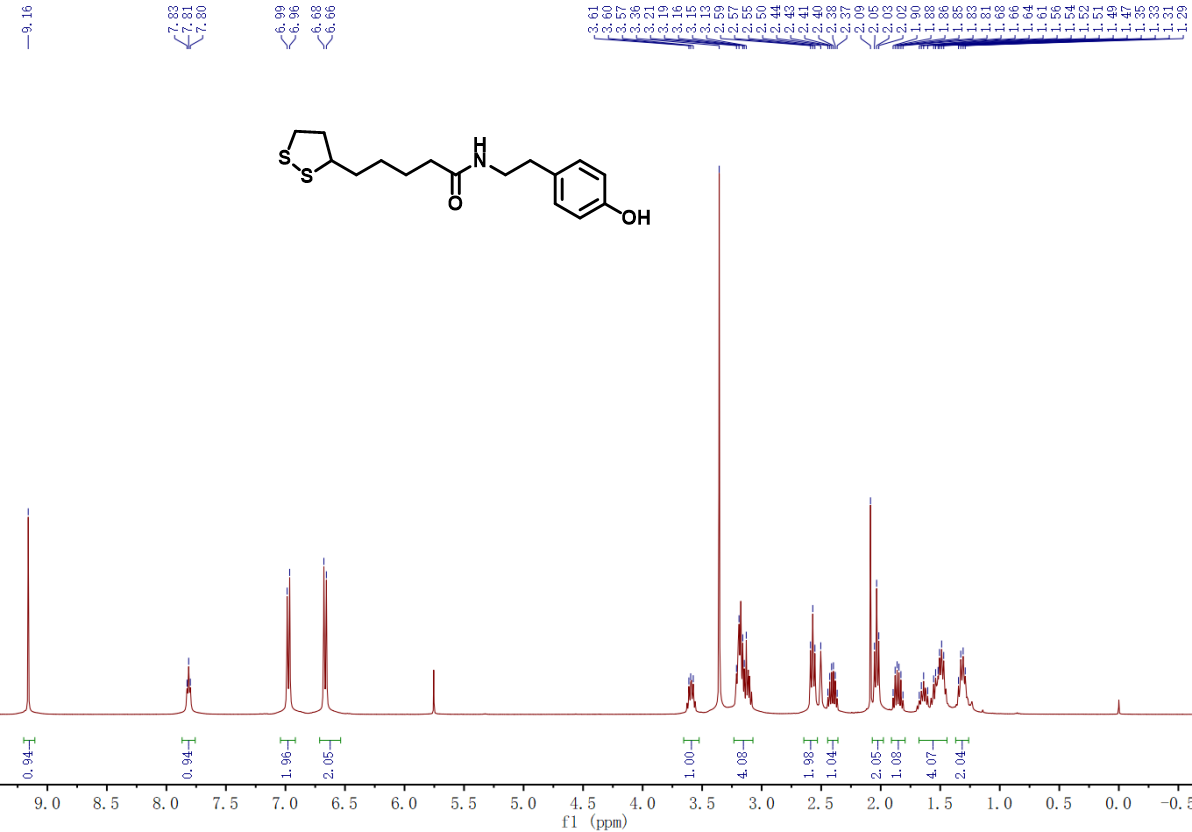
**

**Figure S4.** ^1^H NMR spectrum of compound TAP (400 MHz, 298 K, DMSO-*d_6_*).

**Figure S5.** ESI-mass spectrum of compound TAP ([M+Na^+^]^+^: 348.1069).

**
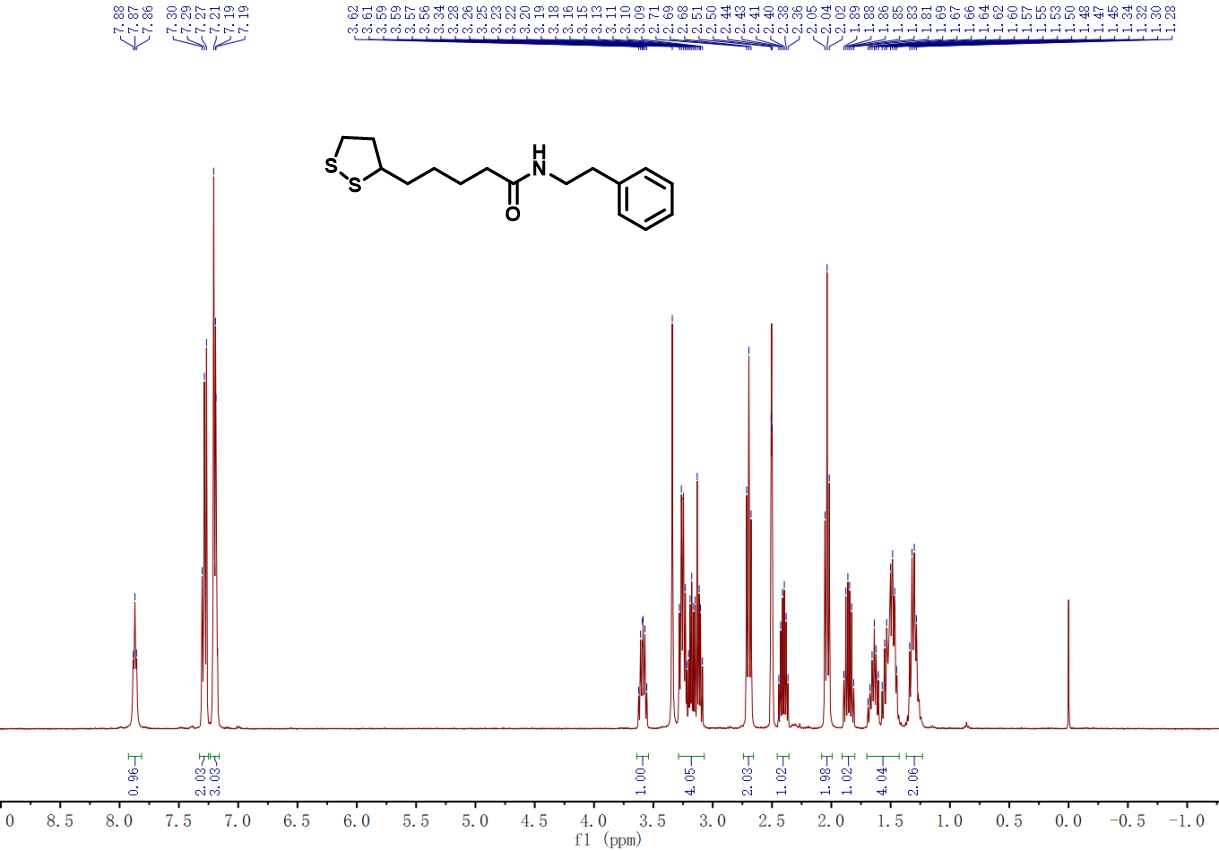
**

**Figure S6.** ^1^H NMR spectrum of compound TAB (400 MHz, 298 K, DMSO-*d_6_*).

**Figure S7.** ESI-mass spectrum of compound TAB ([M+Na^+^]^+^: 332.1118).


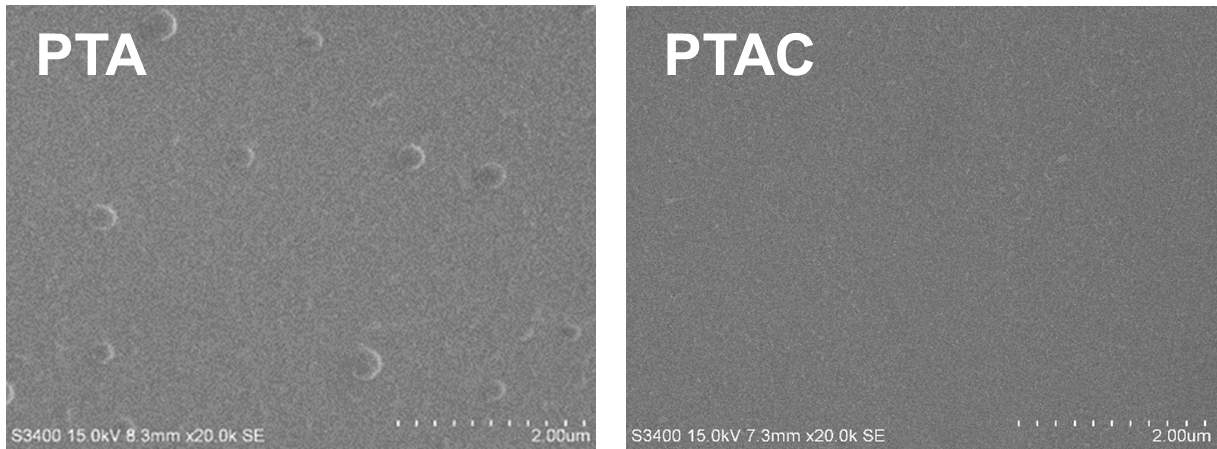


**Figure S8.** SEM images of the PTA and PTAC copolymer. (Scale bar is 2.00 μm.)

**
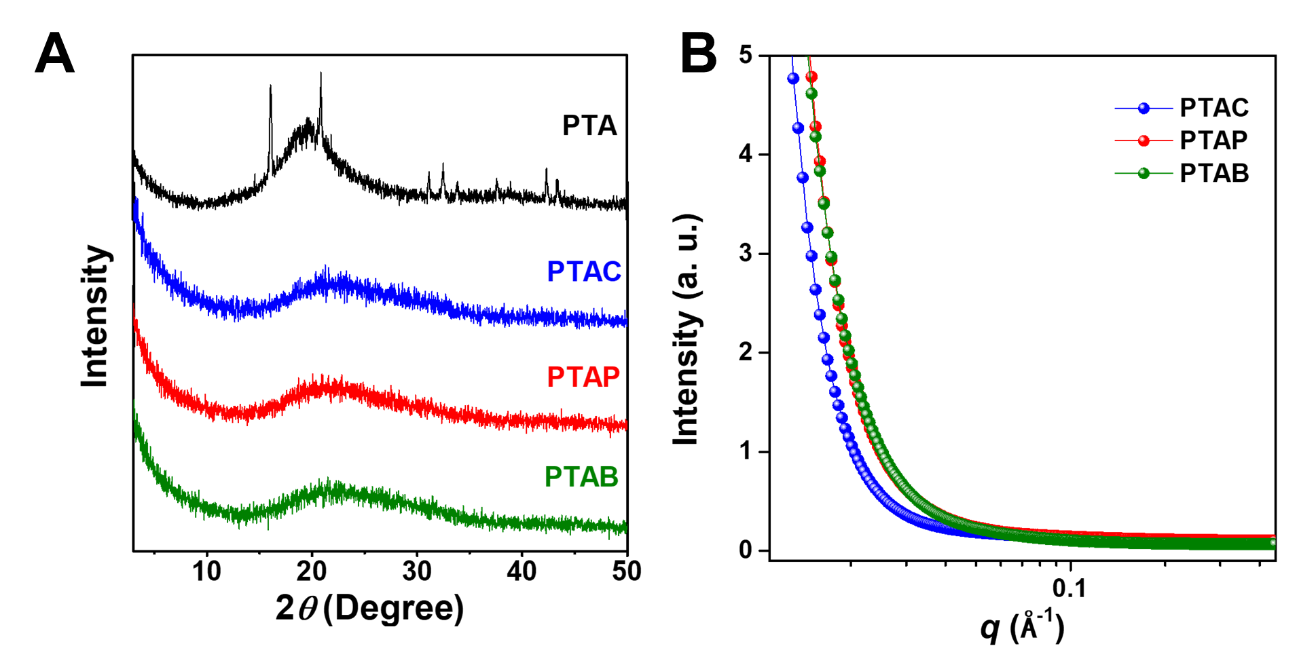
**

**Figure S9.** (A) XRD and (B) SAXS spectrum of the PTAC, PTAP and PTAB copolymers.

**Figure S10.** Temperature dependence of viscoelastic shift factors a_T_ of master curves at a reference temperature of 20^o^C.

**Figure S11.** Underwater shear strength histogram of the PTAC copolymer with different TA and iron (III) concentrations.

**
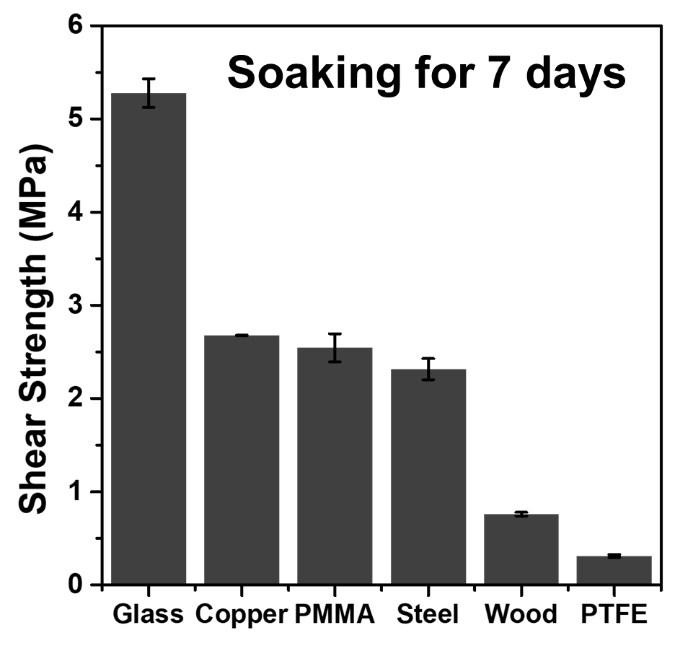
**

**Figure S12.** Comparison of underwater shear strength of the PTAC copolymer on different substrates soaking for 7 days.

**
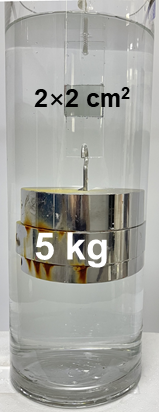
**

**Figure S13.** Photograph of the PTAC copolymer bonded glass sheets to lift weights of 5 kg with a bonding area of 2×2 cm^2^.

**
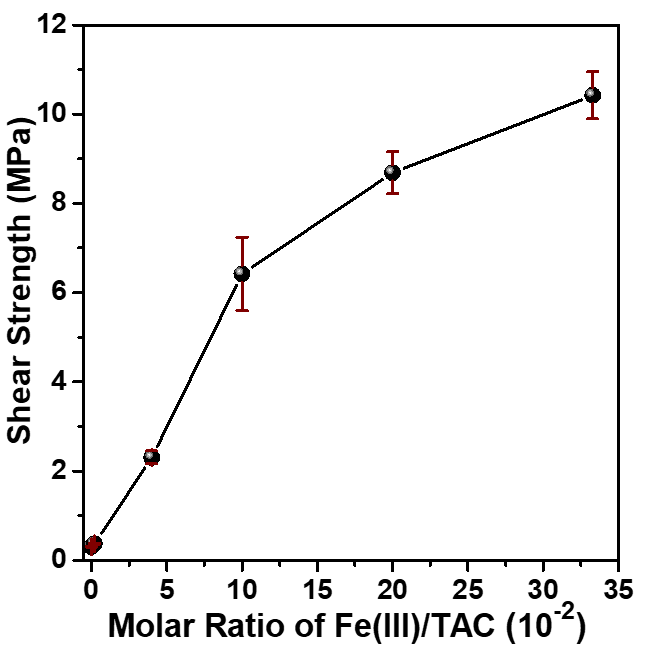
**

**Figure S14.** Shear strength of the PTAC copolymer for different TAC-to-iron (III) molar ratios.

**
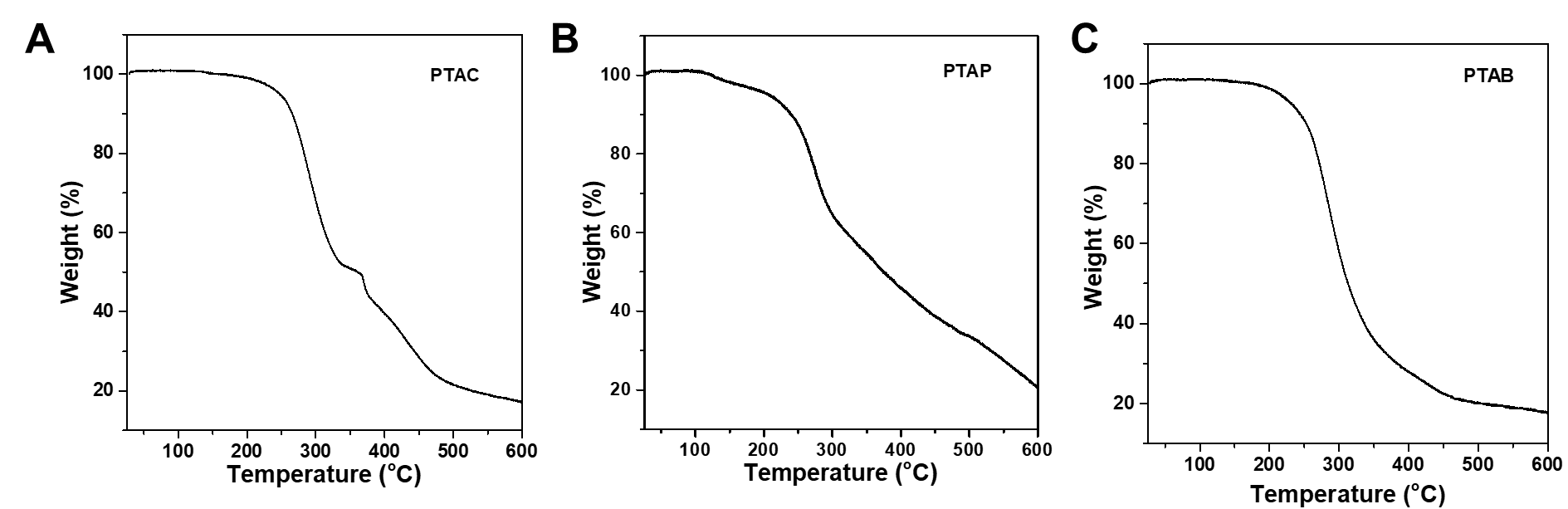
**

**Figure S15.** TGA of the PTAC, PTAP and PTAB copolymers.

**
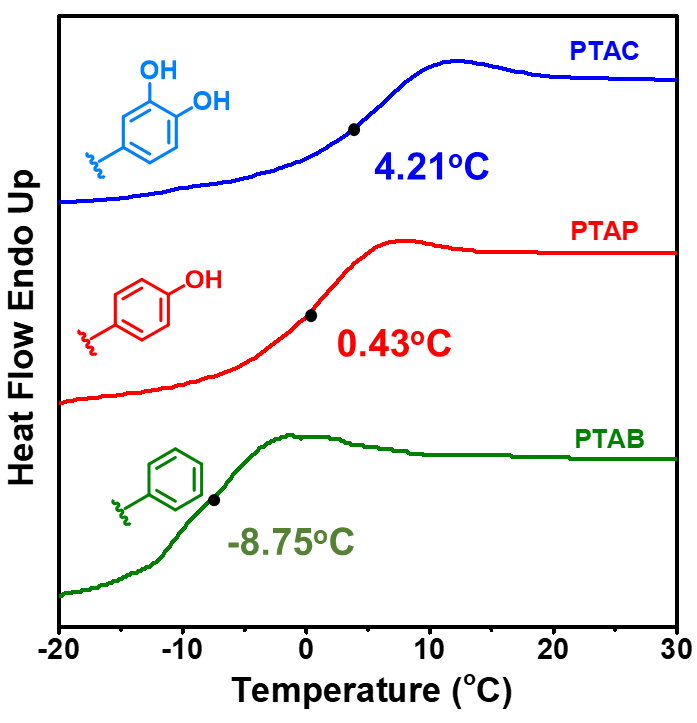
**

**Figure S16.** DSC of the PTAC, PTAP and PTAB copolymers.

**
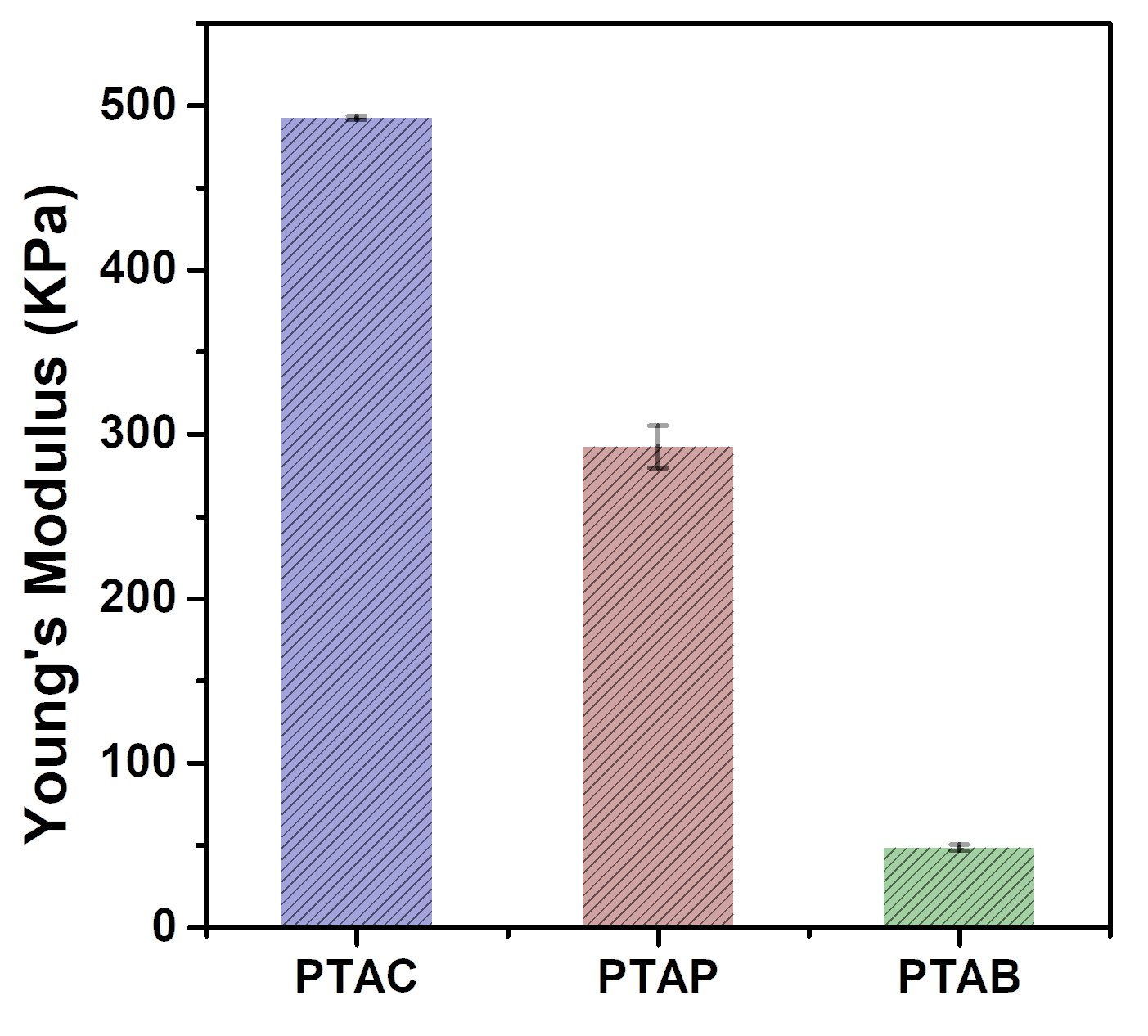
**

**Figure S17.** Young’s Modulus of the PTAC, PTAP and PTAB copolymers.

**
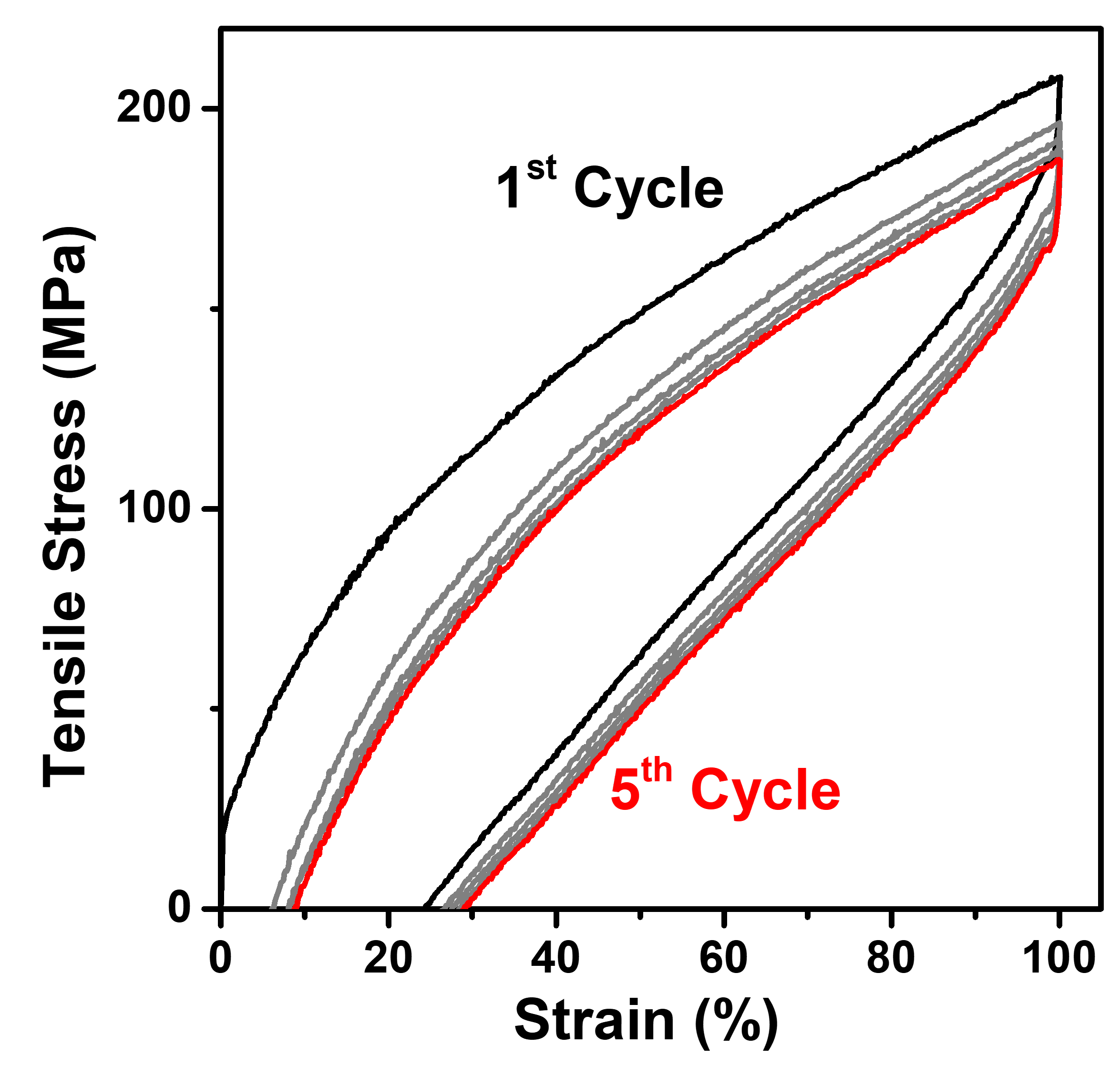
**

**Figure S18.** Five successive loading-unloading cycles of the PTAC copolymer at a loading rate of 20 mmmin^-1^.

**
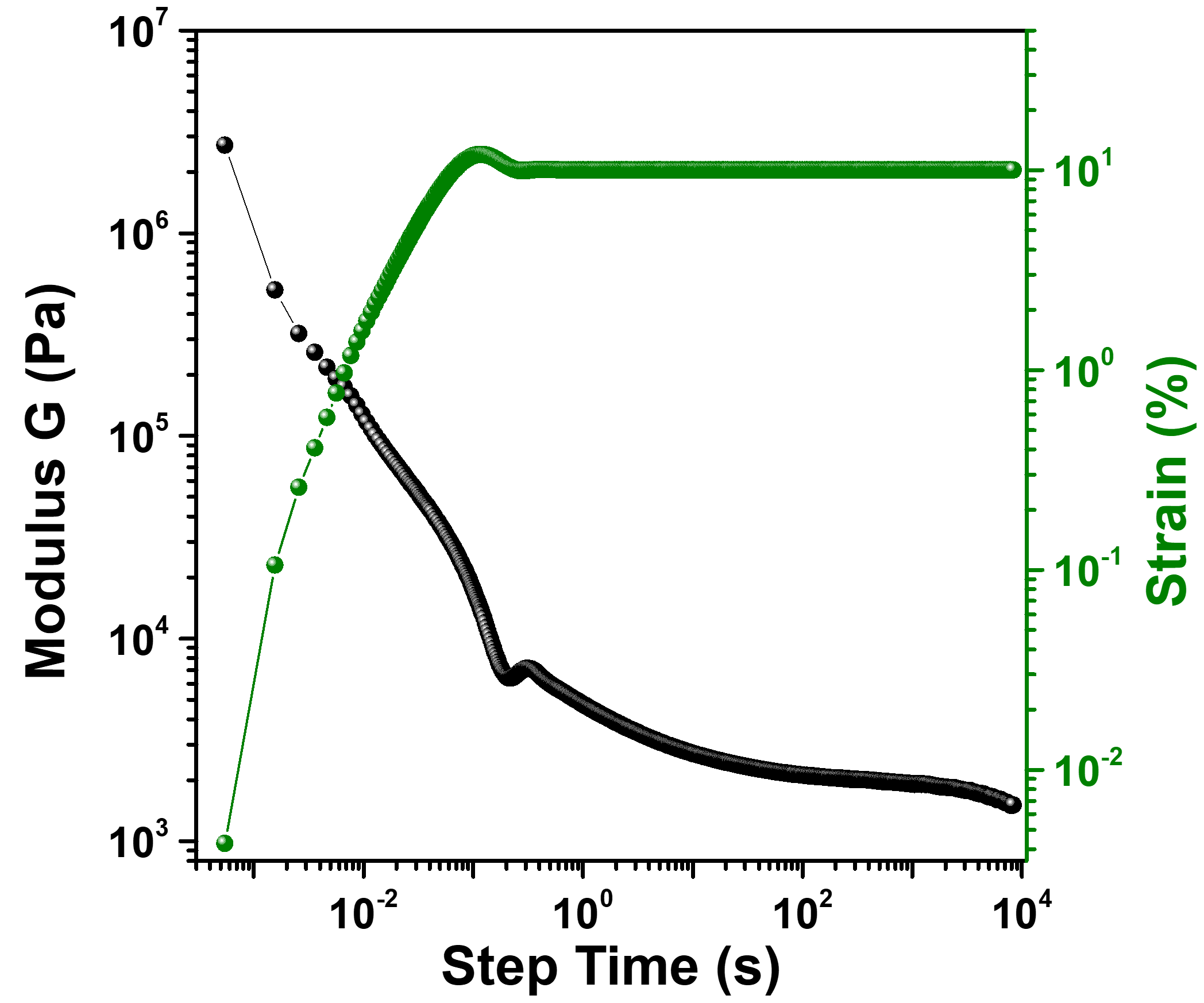
**

**Figure S19.** Stress relaxation experiment performed on the PTAC copolymer. The relaxation strain was 10%.

**Figure S20.** Creep experiments at varying stress were performed on the PTAC copolymer.

**
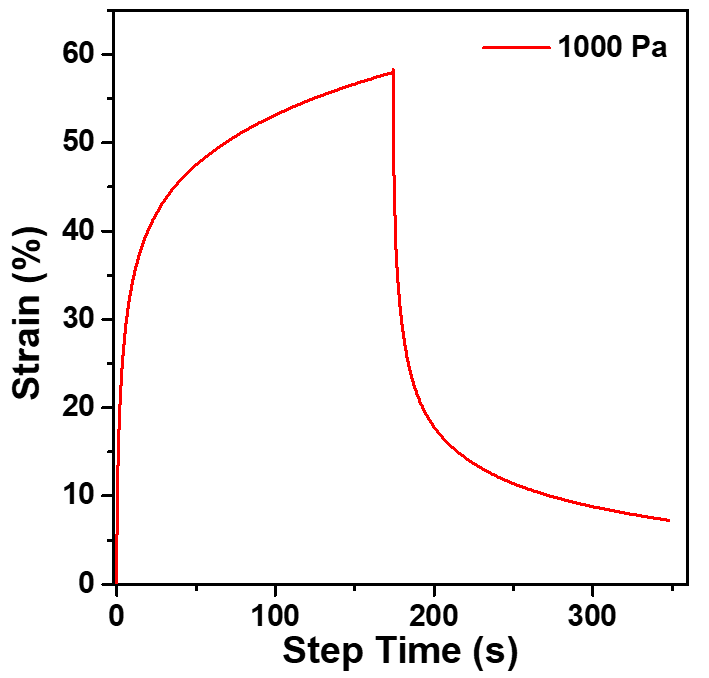
**

**Figure S21.** Creep recovery curves of the PTAC copolymer.

**
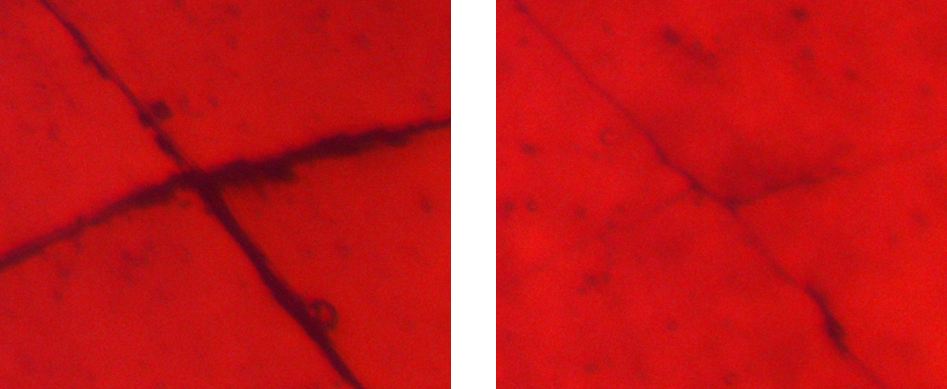
**

**Figure S22.** Photographs depict that scratches on the PTAC copolymer membrane can be autonomously cured after 24 hours at room temperature.

**
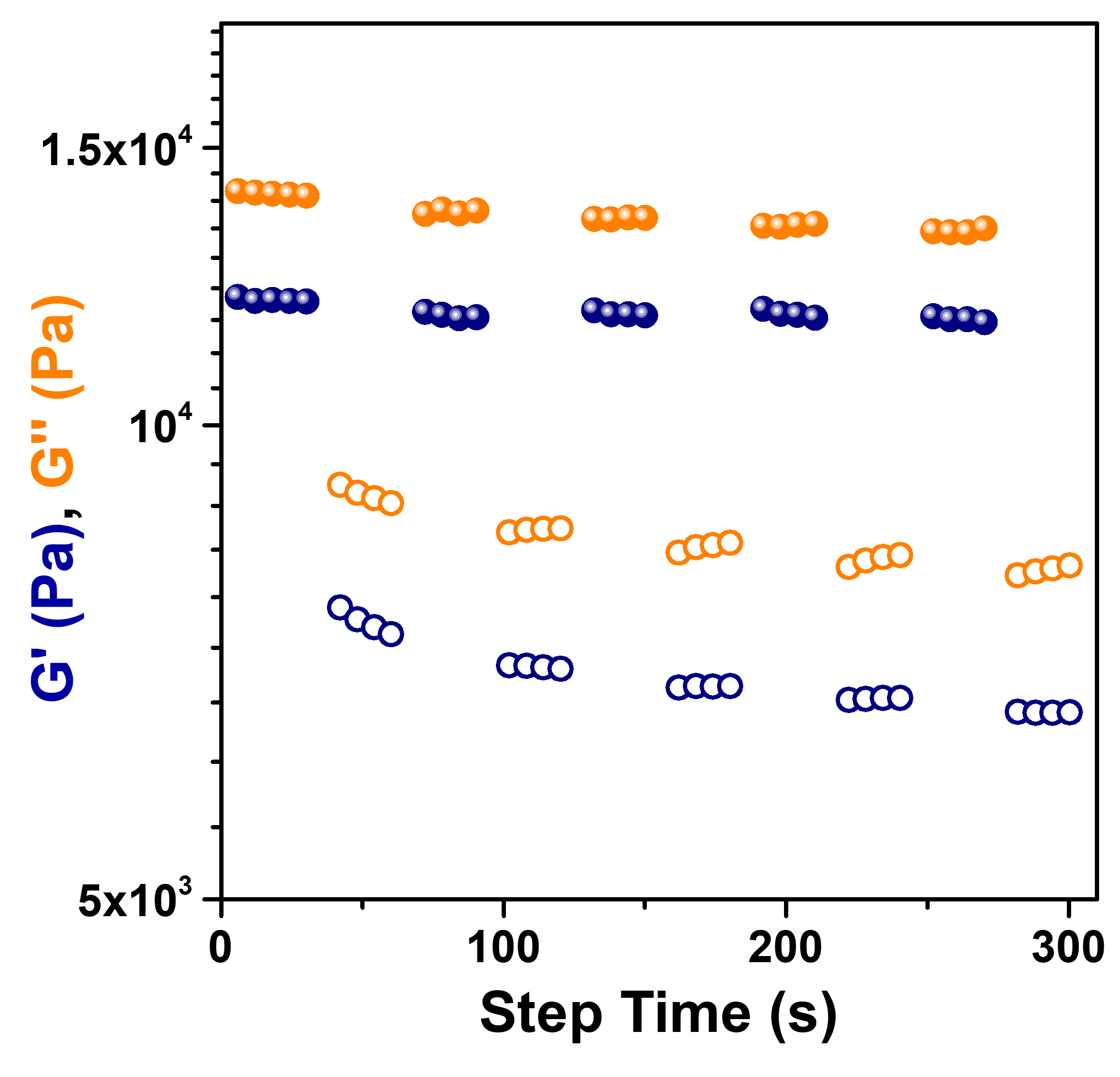
**

**Figure S23.** Storage (G′) and loss (G″) moduli variation under continuous strain sweep variation with small oscillation (0.1 % strain, solid line) and large oscillation (40 % strain, dot line) force alternatively. Angular frequency = 1 Hz.

**
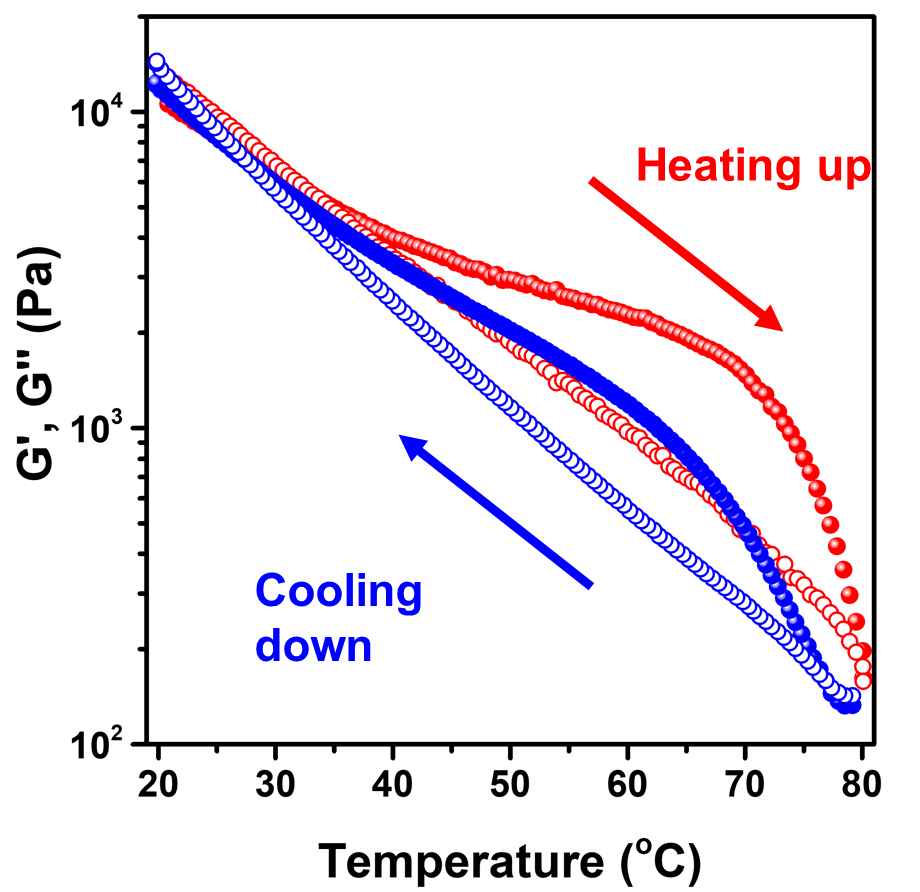
**

**Figure S24.** Temperature-cycle rheology curves of the PTAC copolymer.

**
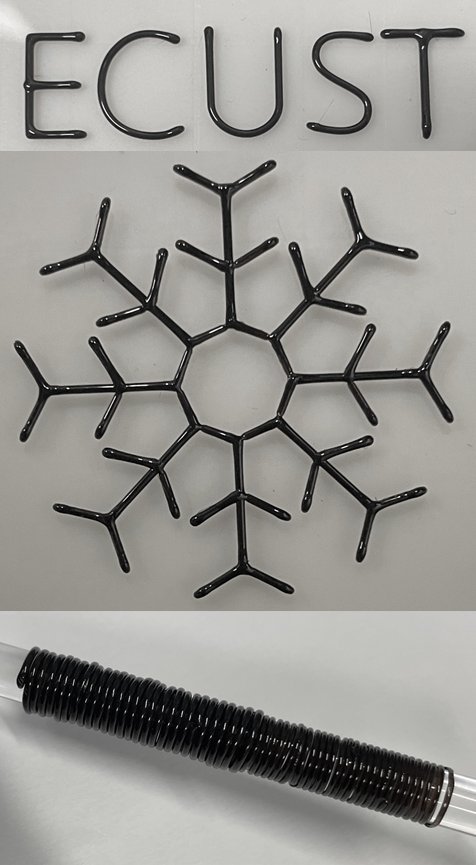
**

**Figure S25.** Photographs of 3D-printing with specific shapes and sizes.
